# Implants in disabled patients: A review and update

**DOI:** 10.4317/medoral.19564

**Published:** 2014-03-08

**Authors:** María J. Romero-Pérez, María R. Mang-de la Rosa, Julián López-Jimenez, Javier Fernández-Feijoo, Antonio Cutando-Soriano

**Affiliations:** 1 DDS, Collaborator of the department of Dentistry in Special Patient. University of Granada; 2 Private activity. Barcelona; 3 Profesor Associated of Dentistry in Patient Special. Group of Investigation OMEQUI. University of Santiago of Compostela; 4 PhD,MD,DDS. Department of Dentistry in SpecialPatient. University of Granada

## Abstract

The range of indications for dental implants has broadened enormously owing to their predictability and the improvement of patient satisfaction in terms of stability, comfort, aesthetics and functionality. The aim of this article is to review those indications in patients with mental or physical disabilities as the difficulty to cope with oral hygiene often leads to teeth extraction, adding edentulousness to the impairments already present. Following that goal, available literature in Pubmed database, Scopus, Web of Knowledge and The Cochrane Library database about dental implants placement in these patients has been reviewed, assessing the variables of each study: number of patients, sex, average age, oral hygiene, parafunctional habits, impairment, bone quality, protocol of implant surgery, necessity of deep intravenous sedation or general anesthesia, follow-up period and number of failures. The comparison with studies involving other patient populations without mental or physical impediments did not show statistically significant differences in terms of the failure rate recorded. Although there is not much literature available, the results of this review seem to suggest that osseointegrated oral implants could be a therapeutic option in patients who suffer from any physical or psychological impairment. The success of an oral rehabilitation depends mainly on an adequate selection of the patients.

** Key words:**Implants, disabled, sedation.

## Introduction

Implantology has demonstrated itself to be a useful procedure in dentistry which has allowed the oral rehabilitation of totally and partially edentulous patients for more than 30 years (1), improving this way the stomatognathic system’s comfort and functionality (2). Patients who suffer from mental or physical disabilities used to be excluded because some local and general conditions that they present contraindicated, apparently, the use of implants as part of their dental treatment. However, these patients are in great need of an oral fixed rehabilitation owing to the fact that the neurologic impairment, neuromuscular disorders, genetic syndromes or orocraniofacial anomalies involve more frequency of dental agenesis (3) and the difficulty of ensuring adequate oral hygiene (4). Moreover, if they wear removable prostheses, their manipulation and hygiene may pose some difficulties (5). When conventional fixed prostheses cannot be placed, an implant-supported rehabilitation may be the only solution.

With regard to the local conditions, daily bruxism has been found to be common in children with brain damage (6,7), which is a risk factor and a relative contraindication to implant placement (8,9), as well as a poor oral hygiene for soft tissue, especially relevant when O’Leary plaque index is over 20%, being this last condition a general absolute contraindication for implant rehabilitations despite the controversy generated by the results found in other studies.

Regarding general conditions, most of these patients must be treated under anxiolytic premedication, deep intravenous sedation or general anesthesia, depending on the degree of cooperation and the difficulty and duration of the treatment provided, as we usually find a total lack of cooperation from these patients (10).

## Material and Methods

This review is based on the research of articles about the placement of implants in patients who present some physical or neurological disability in Medline database (Pub-Med), Scopus, Web of Knowledge and Cochrane between 1992 y 2012 using the key words “dental implants” “disabled patients” and “handicapped people” in different combinations. Some other relevant articles were found in the references of the first ones.

## Results

There has been considered inclusion criterion for this revision any disease involving disability, either physical or mental; and systemic diseases which do not include that sort of disability have been excluded. After a deep analysis of studies carried out in patients with different types of disability, only six of them fulfilled the inclusion criterion. Between the most common disorders were: Down syndrome, fragile x syndrome, autism, schizophrenia, Rett syndrome, cerebral palsy, head injuries, pyknodysostosis, Rieger syndrome, senile dementia etc.

The articles finally selected were published in the following dental journals: International Journal of Oral maxillofacial implants, Journal of Oral Implantology, Special Care Dentistry, International Journal of Prosthodontics, Brazilian Dental Journal, Dental Update and Oral medicine, among the most representative ones.

## Discussion

It has not been possible to perform a meta-analysis nor to provide recommendations based on conclusive scientific evidence, given the lack of long-term randomized studies and relatively small sample sizes. The articles selected are explained below, assessing their most important variables.

Anders Ekfelt (11) carried out a prospective study in patients with neurological disabilities between 2000 and 2003. 35 implants were placed in 14 patients through a standard protocol in two phases. Those patients had one or more of these diseases: Down syndrome, fragile x syndrome, autism, schizophrenia, Rett syndrome, as well as all the medications to treat them and their side effects which are also part of implants’ indication or contraindication. No bruxism was observed in 2 people, 9 were categorized as having little bruxism and 3 of them had sometimes strong bruxism. Bone quality was also recorded according to Lekholm and Zarb as B2 (3 patients), B3 (4 patients) and C3 (7 patients). Implants were placed under general anesthesia in 11 patients, and with local anesthesia in 3 patients. Each implant received a prognostic score from 1 to 4 (1=uncertain;4=very good). The higher prognostic score required good bone quality (7 implants were not placed in bone with good quality), good initial stability (1 did not have it), no exposed threads (9 did not have it) and placement done according to the standard protocol. All patients and their caregivers were given an individual prophylactic program and their oral hygiene was checked up every three months when possible. The observational period after placing the prostheses was between 6 and 28 months, working out the cumulative survival rate through life table analysis. A total of 5 implants failed, 2 of them with a prognostic score of 3 and 4 in one patient with Down syndrome, which results into a 80.5% survival rate. The failures in this patient can be associated with inmunosuppression (12,13).

Such a high rate of survival seems contradictory owing to the frequency of parafunctions such as bruxism, inadequate bone quality or exposed threads, as well as the fact that the two implants that failed are those with the highest prognostic score. However, as the author points, this can be associated with the tendency towards infection from this patient, which caused rapid bone loss in one implant and a sequestration in the other one. The survival rate does not distance from the one found in general population and consequently, implants can be a suitable option in these patients.

López Jiménez and col (14) made a study between 1992 and 2001 in which 67 implants were placed in 18 patients from 12 to 71 years old who had the following diagnosis: cerebral palsy, head injuries, pyknodysostosis, Down syndrome, Rieger syndrome and senile dementia. The surgical procedure required general anesthesia in 9 patients, deep intravenous sedation in 6 patients and anxiolytic premedication in 3 patients. Between 5 and 8 months were waited for loading the implants in the maxilla and 3-4 months in the mandible. They had been loaded an average of 66.5 months (between 3 and 113 months) when they were evaluated. The lack of clinical symptoms and mobility were considered successful criteria as well as the absence of radiotransparencies when radiological follow-up was possible. 4 implants failed during the osseointegration period in 3 patients (all with genetic syndromes: two patients with Down syndrome and one with Rieger’s syndrome). 3 of the 4 implants were placed in the only two males with Down syndrome of the sample, in the incisive region of the maxilla, which can be associated with the higher frequency of periodontal disease in these patients (15). All of them could receive a successful rehabilitation with fixed prostheses.

This is a study with a long follow-up period and a sufficient sample size including different types of impairment which allows us to compare it with non-disabled samples, though we also have to consider the bias produced by the heterogeneity of the sample. Bone quality or parafunctions are not specified, which may be relevant, but as opposed to other studies, the location of unsuccessful implants is mentioned. As a result of this study, it seems that implantological treatment is possible in these patients, as no major differences are found when compared to patients without discapacity.

The inclusion of patients with Down syndrome seems to be the most frequent one in the studies reviewed despite the fact that implatological treatment has been long questioned owing to the high rate of some disorders such as osteoporosis, macroglossia, occlusion problems (16), parafunctions, periodontal disease (15), poor oral hygiene and cooperation, inmunosuppression (12,13) etc. However, the frequency of microdontia in permanent dentition (17,18), hypoplasia, hypodontia (19), altered crown morphology (20) and the increased life expectancy of these patients obliges to assess a new method of treatment which has already been studied by some authors. Those cases are explained below.

Ribiero and col (21) published a case report in which 7 maxillary implants and 5 mandible implants were placed in a 36 year-old woman with Down syndrome. The patient had moderate mental retardation but was able to speak and to perform simple everyday tasks without difficulties, for this reason it was not necessary to use auxiliary techniques such as general anesthesia or deep intravenous sedation. She had a poor oral hygiene and periodontal disease (moderate bone loss in the maxilla and severe bone lost in the mandible). 6 months after the placement of the implant, the radiographic assessment showed peri-implant bone loss around dental implant number 22, as well as pain. For those reasons, it was not included into the rehabilitation. The rest of the implants were successfully osseointegrated.

Soares and col (22) also published a case report about the success of an implant in a patient with Down syndrome. This patient had moderate mental retardation, pseudomacroglossia and sleep obstructive apnea syndrome. A maxillary left central incisor was replaced with the help of general anesthesia and was immediate loaded. The patient was followed up clinically and radiographically for 4 years without showing any sign of failure.

Additionally, Lusting and col (23) made a study in a 16 year-old patient with Down syndrome and partial anodontia, who received 4 implants in the place of 15,25,34 and 45. The patient had moderate mental retardation, gingivitis, dental plaque, macroglossia, anterior open bite and hypersalivation. The surgical procedure required intravenous sedation. Bone was very spongy and for this reason a gradual loading was performed for one year, starting 8 months after their placement. Implant number 34 failed and this was attributed by the authors to its narrower diameter. Osteoporosis bone was not a risk factor, as it is not in general population (24,25).

Rogers (26) studied the response from a patient who suffered from athetoid cerebral palsy towards the implantological treatment necessary to place a mandibular implant overdenture. The patient was 64 years old and her bone quality and quantity was adequate for implant surgery. Her dental hygiene was good but she presented involuntary movements of the mandible, tongue and lips which led to incapability to wear the prostheses. 4 implants were placed in the canine and premolar regions of the mandible with the help of general anesthesia to control involuntary movements. Finally, the prostheses was placed over two implants, leaving the other two submucous, and obtaining very positive results because of the improvement in speech and chewing.

The response from patients suffering from Parkinson’s disease towards the implantological treatment has also been studied (27), being this one pretty satisfactory. Implant supported prostheses provide stability, make easier to insert, remove and clean the prostheses and reduce gastrointestinal problems because of the improvement in chewing function.

The  Table 1 shows all the studies reviewed and the results in terms of failure.

**Table 1:**
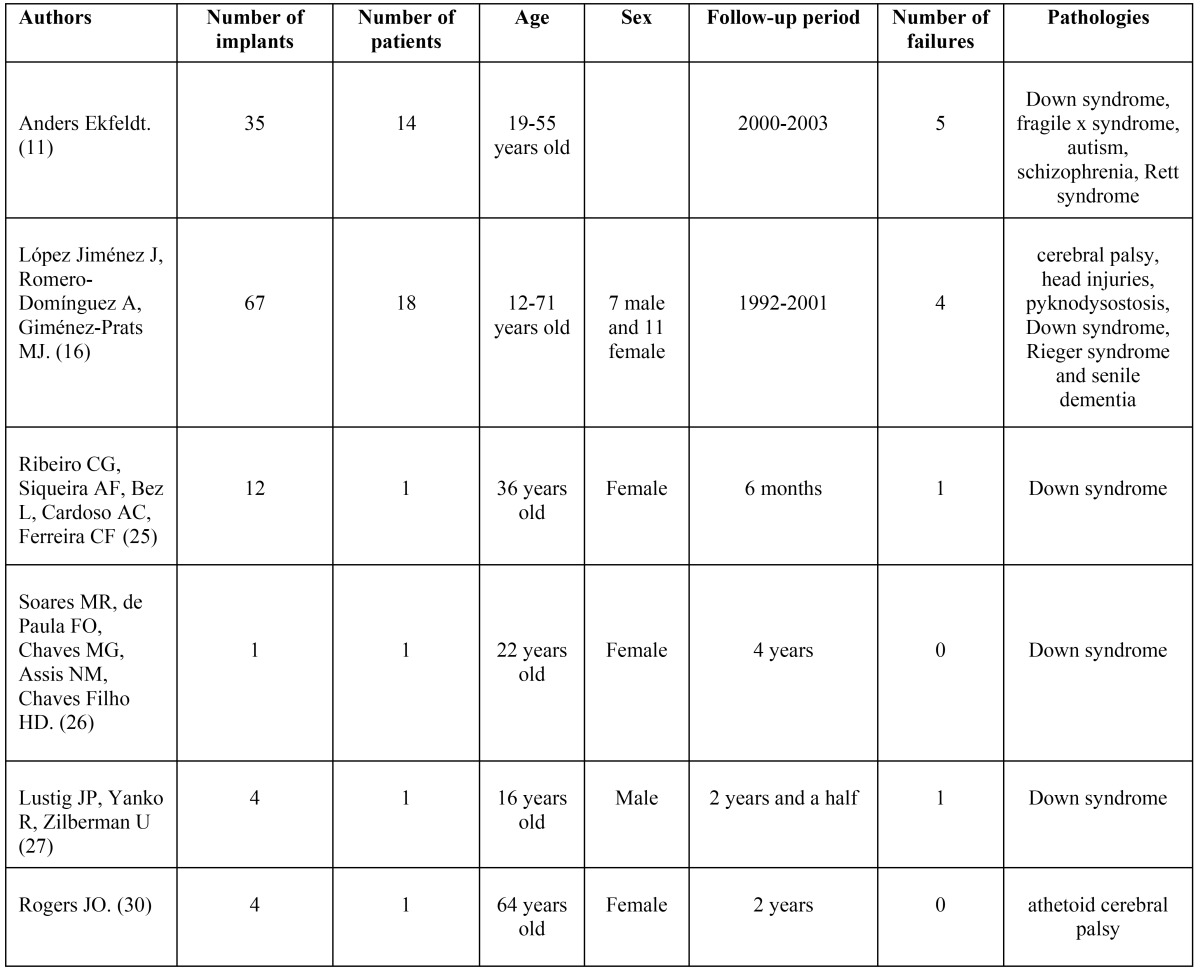
Studies reviewed and their results in terms of failure.

## Conclusions

More studies with bigger sample sizes and further follow-ups should be carried out, with detailed information about the systemic condition of each patient, the presence of parafunctions, hygiene and placement of the implants ( anterior or posterior region of the maxilla or the mandible). This last issue is especially relevant because bone quantity and quality are decisive factors in the survival of implants, and very few authors mention this variable as it has been shown. The comparison with studies involving other patient populations without mental or physical impediments did not show statistically significant differences in terms of the failure rate recorded.

Oral health is an integral part of general health and for this reason it must be reestablished when it is altered, especially in those patients who have the greatest need, providing them all the resources of modern Dentistry so as to improve oral function and aesthetics, regardless of their physical or neurological condition. It is necessary to evaluate each case individually, following a strict surgical protocol and frequent checkups, as well as informing the patient’s caregivers about the importance of maintaining good oral hygiene and the absence of oral habits.

It must be kept in mind that edentulousness is frequent among disabled patients and implants may be the only choice that not only reestablishes oral health but provides an increase in patient self-esteem from an aesthetic point of view as well as in their quality of life, reduced by other diseases.

Although more experience is needed, implant rehabilitation can be considered a suitable option in people with disabilities as bone quality and quantity seem to be more relevant in order to achieve positive outcomes.
